# The METEMP protocol: Massed exposure therapy enhanced with MDMA for PTSD

**DOI:** 10.1016/j.conctc.2024.101400

**Published:** 2024-11-24

**Authors:** Jessica L. Maples-Keller, Boadie W. Dunlop, Barbara O. Rothbaum

**Affiliations:** Emory University School of Medicine, Department of Psychiatry and Behavioral Sciences, Georgia

**Keywords:** MDMA, PTSD, Clinical trial, Exposure therapy, Treatment, Psychedelic

## Abstract

This article describes the rationale and the specific methods for an open label pilot trial of 100 mg MDMA in combination with massed exposure therapy for PTSD, a treatment which involves daily exposure therapy sessions for two weeks which has found to be effective. We review the need for novel PTSD treatments and the existing research on MDMA-assisted therapy, and then describe the rationale for this novel treatment approach, including combining MDMA with a gold standard treatment, translational support for this treatment, strong dissemination potential, and strengths of providing massed exposure treatment. The specific methods of the open label pilot study are presented, followed by conclusions and future directions for this research. This study will enroll at least 15 adults with PTSD over the next 2 years in order to identify feasibility and lead to a manual describing how to combine MDMA with exposure therapy, tested in a randomized, placebo-controlled trial, with significant promise for dissemination and improving our ability to treat PTSD.

**Trial registration:**

ClinicalTrials.gov identifier: NCT05746572.

PTSD presents a significant public health issue with a prevalence of 5 % in representative samples [1] and 23 % in recent veterans [2]. Current PTSD clinical practice guidelines recommend trauma focused therapy such as Prolonged Exposure (PE) or Cognitive Processing Therapy (CPT) as first line treatments [[3], [4], [5]]; however, it would be beneficial to decrease attrition and many patients do not achieve meaningful symptom improvement [6], indicating the need for novel treatment interventions.

3,4-methylenedioxymethamphetamine (MDMA, “ecstasy”) is a ring-substituted phenethylamine with structural similarities to methamphetamine and mescaline which is associated with subjective effects such as euphoria, empathy, insightfulness, and feelings of peace or well-being [7,8]. In two recent Phase III trials, MDMA-AT for PTSD resulted in significantly greater reductions in PTSD symptoms compared to placebo plus therapy at post-treatment (*d* = .91; d = .7)^11^. The therapy model used in these trials has not previously been tested as a standalone PTSD therapy/is not an evidence based existing PTSD therapy. The focus of the treatment is on the participants’ “inner healing intelligence,” or their “innate capacity to heal the wounds of trauma” [9]. This model is reflective of historical models of MDMA therapy which were largely non-directive [10] and focus on a model of MDMA as the treatment and the therapeutic component supporting this. Our study will focus on a different model in which MDMA is used as an augmentation strategy for an active, evidence-based treatment.

The current paper provides the rationale and methods overview for our investigation of a novel treatment approach: Massed Exposure Therppapy Enhanced with MDMA for PTSD (METEMP), combining MDMA with an evidence-based therapy for PTSD. First, we provide the rationale for several aspects of this novel approach, including the use of exposure therapy, a massed approach, investigating fear extinction as a mechanism, and for the open label pilot methods. Massed exposure therapy is a model in which exposure therapy is provided daily for two weeks, instead of the usual once per week for several months’ treatment approach. Next, we provide an overview of the methods for this open label clinical trial, followed by a description of future directions of this research.

## 1 Prolonged exposure therapy: a gold standard PTSD treatment

PE is a gold standard PTSD intervention [3,4] with a significant body of research finding it highly effective [11]. Within PE, patients approach feared stimuli in a systematic, gradual, and therapeutic manner via imaginal exposures, in which patients recount the trauma memory aloud and in their imagination during therapy sessions, and in vivo exposures, in which patients approach safe trauma reminders in real life. MDMA has not previously been investigated with a gold standard PTSD intervention.

## 2 Translational support

PTSD is characterized by experimental extinction learning and recall deficits [12,13]. Within this fear conditioning characterization of PTSD, exposure to a trauma represents an unconditioned stimulus (US) that elicits an unconditioned fear response (UR). The environmental cues present at the time of trauma are then associated with the US (trauma) and thus serve as conditioned stimuli (CSs) and acquire the ability to produce subsequent conditioned fear responses (CRs). In the laboratory, fear extinction training involves repeated presentations of the CS without the US, resulting in a decreased conditioned fear response [14]. Fear extinction recall is the retrieval and expression of the learned extinction memory following a delay. PE is based on emotional processing theory and fear extinction learning-treatment techniques include helping patients approach trauma-related stimuli in a therapeutic manner resulting in decreased distress and fear responses over repeated exposures (i.e., fear extinction).

MDMA administration before extinction training has been shown to enhance fear extinction of conditioned freezing in mice [15]. The enhancing effect of MDMA on fear extinction was replicated and extended using fear potentiated startle in another study in which a serotonin reuptake inhibitor was found to block this effect in rodents, suggesting the importance of serotonergic neurotransmission in this process [16]. A recent randomized, placebo-controlled trial in healthy adults tested in a fear extinction paradigm found that significantly more participants in the MDMA group were extinction retainers compared to the placebo group [17], providing support that MDMA may facilitate retention of extinction learning and thus could be a beneficial enhancement strategy for PE for PTSD. Empirical evidence supports that experimental extinction learning is relevant to actual PE clinical outcomes. In a sample of veterans receiving massed PE, high responders (i.e., greater than 50 % reduction in PTSD symptoms) demonstrated significant extinction retention learning, whereas low responders showed a distinct return of fear [18], supporting that extinction retention is relevant to massed PE treatment response, and supporting the investigation of MDMA's impact on fear extinction as a possible mechanism of PE enhancing effect.

## 3 Dissemination potential

This is a novel treatment method and significant additional research is needed in order to establish its safety and efficacy-if successful, the proposed PE + MDMA treatment would have a profound impact on optimizing treatment for PTSD with strong dissemination potential [19]. VA medical centers are required to provide access to either PE or CPT for PTSD, as such there is a large number of trained providers competent in these interventions. Within the VA system, many models of providing PE and CPT exist, including outpatient, massed treatment, and residential treatment-providing different models for which a one-time MDMA administration could be incorporated within ongoing programs, many of which have access to physicians and medical screening and monitoring. The current Lykos MDMA-AT model involves 42 h of treatment with two therapists across approximately three months, with 3 8-h MDMA dosing sessions. This proposed novel model involves a one-time MDMA dosing session plus 9 additional therapy sessions, with the primary therapist present for all sessions and assisting therapist present for the first session, dosing session, and third session. Hence, this model, if effective, would scale well to existing care delivery systems.

## 4 Why massed exposure?

Massed PE [20], in which therapy is conducted two or more times per week compared to a typical one session per week schedule, is non-inferior to traditional spaced PE [21], and gains are maintained over 12-month follow-up [22]. The Emory Healthcare Veterans Program provides the massed PE treatment approach used in this protocol and has treated over 1300 veterans and service members with PTSD, achieving large reductions in PTSD and depression symptoms and a 96.3 % treatment completion rate [23]. In many PTSD trials, 30–50 % dropout rates lead to difficulty disentangling attrition from low treatment response [24]; high retention is needed to investigate enhancement strategies for PE. Additionally, a recent study in mice found that psychedelic drugs reopen a social reward learning critical period and that for MDMA and psilocybin, this critical period remained open for 2 weeks [25], suggesting that this window is optimal for neural plasticity and enhancing therapeutic outcomes. A massed PE approach, maximizing both patient retention and MDMA-related neural plasticity, provides the optimal test of MDMA as an enhancement strategy for PE.

## 5 Open label pilot study

This study will represent the first time that MDMA is used in combination with an evidence-based treatment. Most existing treatment approaches are non-directive, particularly during the MDMA administration session. In contrast, this study will implement imaginal exposures during the MDMA administration session. Given MDMA is associated with potentially strong subjective effects [7,8], a pilot study is needed to focus on treatment development, refining the optimal approach to conducting imaginal exposure while participants experience MDMA's subjective effects, and to assess patient acceptability and tolerability.

## 6 Overview of materials and methods

This open-label treatment development and feasibility study combines 10 sessions of massed PE with daily 90-min sessions, including imaginal and in vivo exposure sessions [20], with one dose of 100 mg MDMA HCl on Visit 2 (∼84 mg MDMA). The study aims to test the protocol for combining MDMA and imaginal exposure, with the primary outcomes on CAPS-5-R^29^ scores at one-month follow up compared to pretreatment. The study was approved iby the local ethics committee, written informed consent will be obtained for all participants, and the study is registered at ClinicalTrials.gov (identifier: NCT000123456) and has received IRB approval at Emory University. Recruitment methods include via the Emory Healthcare Veterans Program, advertisement shared with both veteran and community providers, and participants can self refer via the clinical trials website. We plan to enroll at least 15 participants who complete all study methods, but will continue to enroll until we feel that the treatment protocol and manual are well established for a subsequent randomized control trial.

*Inclusion Criteria.* Participants undergo a phone screen, intake assessment, medication tapering if indicated, re-assessment after tapering if needed, electrocardiogram, labs, drug test, pregnancy test, physical, and psychiatric assessment. *Inclusion criteria* include a current diagnosis of PTSD, 21–70 years of age, and agreement to taper off psychiatric medications prior to study entry. *Exclusion criteria* include a history of or current bipolar I or psychotic disorder, current major depressive disorder with psychotic features, lifetime amphetamine or cocaine substance use disorder (SUD), current moderate or severe SUD, positive drug screen for amphetamine or cocaine, current personality disorder, current serious suicidal risk, uncontrolled hypertension, current or history of any medical condition that would make receiving sympathomimetic drug potentially harmful, or currently engaged in compensation litigation whereby financial gain would be achieved from symptoms of PTSD.

**Fig. 1:**
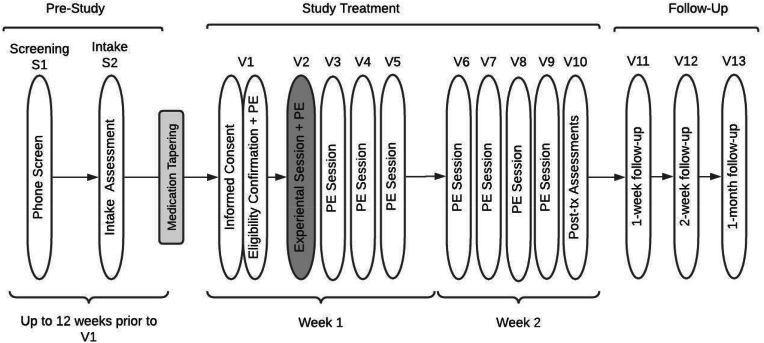
Study Design Note- PE= prolonged exposure.

**Fig. 2:**
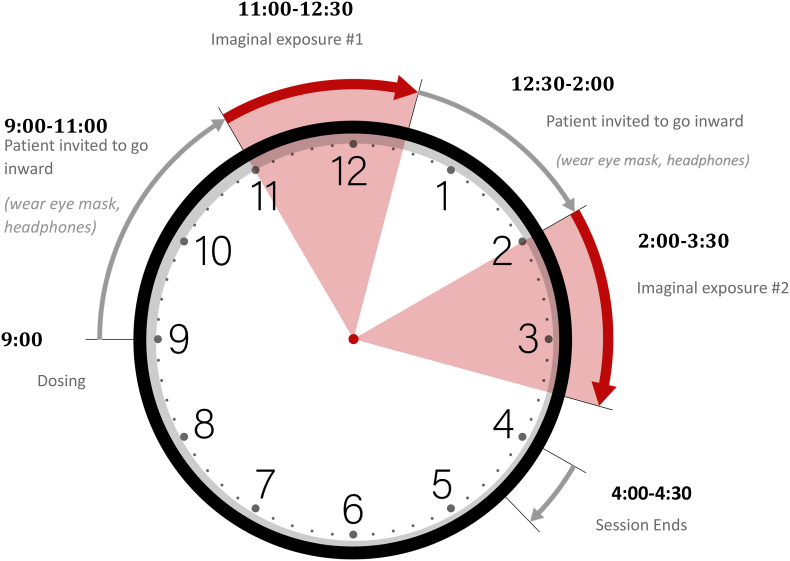
METEMP graphic of dosing day (visit 2).

**Fig. 3:**
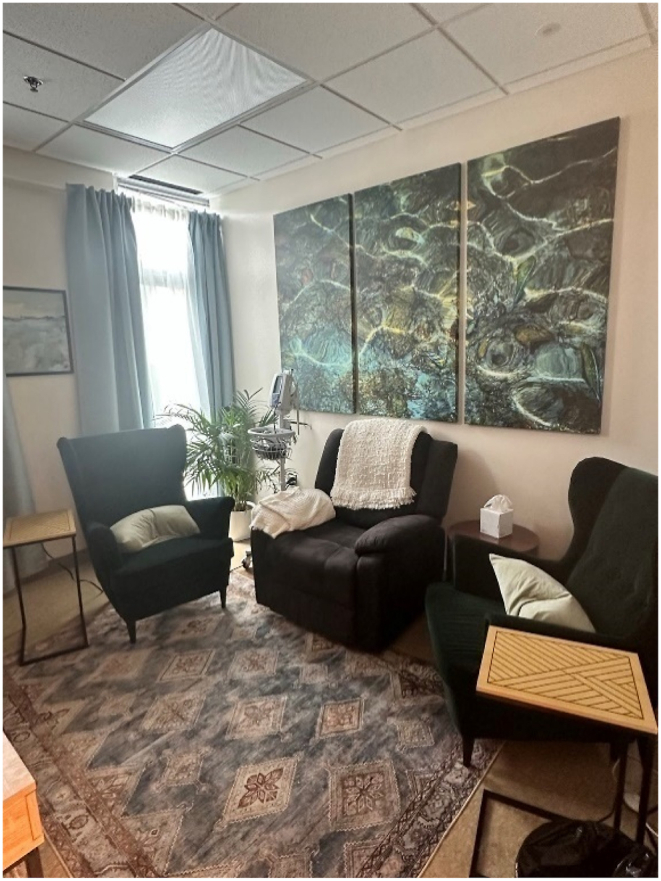
METEMP MDMA room.

**Fig. 4:**
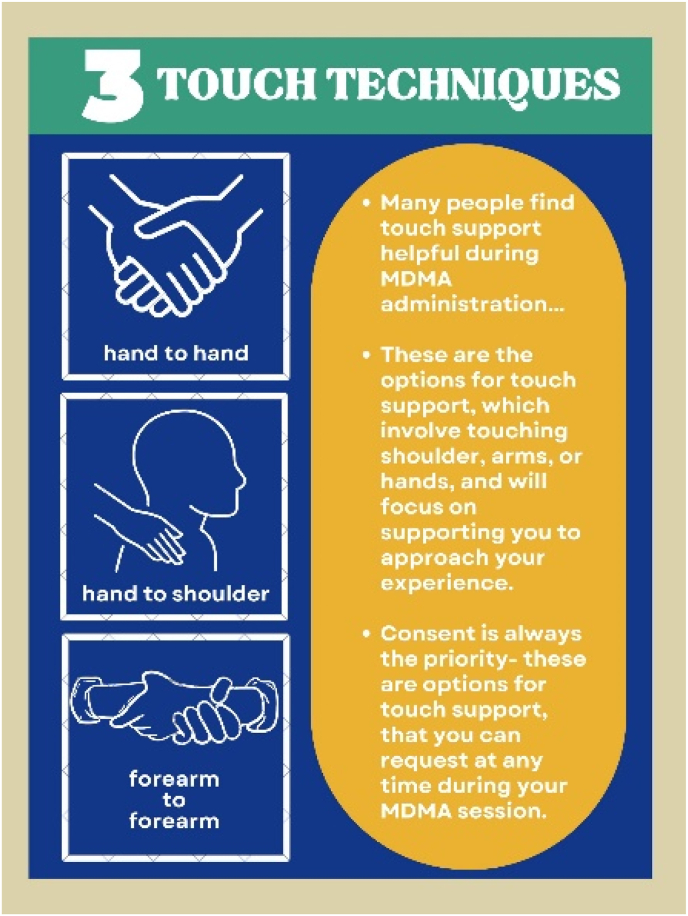
Touch handout.

Treatment Schedule and Session Content. Fig. 1 presents the study design. The initial therapy session (*Visit 1*) incorporates both psychoeducation related to MDMA and the introductory sessions for PE and includes psychoeducation, rationale for exposure-based treatment, rationale for MDMA combined with PE, discussion of Subjective Units of Distress (SUDS) scale, psychoeducation regarding MDMA's subjective effects, breathing relaxation training, and strategies for MDMA-specific support during Visit 2. Sessions 3–10 include standard PE content, including imaginal exposure, processing, and planning and discussing in vivo exposure homework. Fig. 2 shows the timeline of Visit 2 and Fig. 3 is an image of the treatment room. Table 1 presents an overview of the session schedule and content. Because MDMA support strategies can include physical touch, and given the sensitive nature of touch especially with trauma patients, a handout is provided and therapists discuss the available touch options for Visit 2 (Fig. 4). The participant's consent is prioritized and specific ways to request touch or ask for it to stop are practiced during Visit 1. It is emphasized that consent is an ongoing process that is fully the patient's decision at all times, and that touch will always stay within these specific parameters to minimize any risk of harm (e.g., [26]) .

**Table 1:** MDMA + PE summary treatment outline.

Session	Day	Content	Length	Number of therapists
1	1	MDMA + PE preparation: Overview, Rationale, Gather Information, Common Reactions to Trauma, Teach Breathing, Discuss Touch, Review Plan for Visit 2, Decide starting and ending points for the imaginal exposure. Patient given copy of PE workbook.	3–4 h	2
2 & 3	2	MDMA + PE Dosing: going inward, 2 sessions of PE, going inward in between	6–7 h	2
4	3	PE and integration	2 h	2
5–9	4–9	PE, in vivo hierarchy, moving to Hot Spots	90 min	1
10	10	PE- Imaginal Exposure with Entire memory, assess progress, and termination	90–120 min	1 or 2

Note. PE= Prolonged Exposure, MDMA = 3,4-Methyl enedioxy methamphetamine.

“Set and setting” is an important consideration within MDMA-AT [27], meaning the physical environment and expectations and internal factors of the patient impact the MDMA experience. Hence, the MDMA session occurs in a specialized room set up for these sessions (Fig. 3). Specific components include access to natural light, an aesthetically pleasing room with artwork and a calming environment, a comfortable fully reclining chair, and a tailored music playlist and eyeshades for participants to spend time focusing inward when not engaged in an imaginal exposure.

MDMA is administered by the study physician after all safety measures have been completed at 9:00 a.m. Vital statistics are monitored throughout the dosing day (blood pressure and temperature) by the secondary therapist. The first and second imaginal exposures occur approximately 90 and 240 min following MDMA administration, respectively, in which participants close their eyes and describe the index trauma using the present tense with multiple repetitions [28]. The participant reports SUDS before beginning the narrative, after ending the narrative, and the peak during the narrative. Imaginal exposures are recorded for the patient to listen to for homework on a digital recorder. Imaginal exposure is followed by processing in which observations from the imaginal exposure are discussed and made explicit for the patient. At the end of Visit 2, participants are escorted by two study team members to a hotel for an overnight stay, and a well-being check phone call is made later that evening. All study sessions are audio- and videorecorded for fidelity monitoring both for imaginal exposure and PE, as well fidelity to MDMA procedures, and supervision.

Visit 2 aims to be a true combined therapy, allowing time for the patient to “go inward” with the MDMA, and to flexibly begin imaginal exposure when the patient is ready. After MDMA administration, the patient is invited to wear eyeshades and earphones, listen to the study playlist, and go inward for approximately 90 min. There is no expectation of conversation, but if the patient wants to recount what they are experiencing to the therapists, the therapists are receptive. If the patient requests, a breathing technique taught in Visit 1 will be administered. If the patient requests therapist support by touch, therapists provide immediately what the patient requests. When the patient indicates they are ready to begin the imaginal exposure, approximately 90 min after dosing, the eSense electrodes, which measure skin conductance and are used to track physiological responding during imaginal exposure, with electrode gel are attached to the patient's fingers of their nondominant hand. Imaginal exposure proceeds with the patient narrating the traumatic event in the present tense with their eyes closed, adhering to the starting and ending points for the imaginal exposure as decided in Visit 1. Imaginal exposure continues for approximately 45–60 min with as many repetitions of the trauma narrative as that time frame allows.

Immediately following imaginal exposure, processing commences with an open-ended question such as, “How was that for you?” During processing, the patient is invited to make observations. Often this includes remembering more details, which become easier to recount with subsequent repetitions, and how the MDMA experience was for them. In MDMA + PE, depending on the therapists’ judgment, the processing can be accelerated. For example, in standard PE, oftentimes guilt is not addressed until after several sessions of imaginal exposure and processing. In Visit 2, the patient is encouraged to make observations and connections, including to other traumatic events that may have come up for them when going inward, and the therapist can invite discussion of deeper issues such as guilt. Following processing, the patient is invited to wear eyeshades and earphones, listen to the study playlist, and go inward for approximately 60 min. When the patient indicates they are ready to begin the second imaginal exposure, approximately 240 min after dosing, the eSense electrodes with electrode gel are again attached and the second imaginal exposure is begun, followed by processing. The patient is not assigned homework after Visit 2 but is given instructions for self-care. Visit 2 last approximately 6 h. At the end of Visit 2, the study physician meets with the study participant to assess appropriateness for discharge, including psychiatric (i.e., assessing for active suicidality, disorientation, extreme psychologist distress that is not responsive to therapist-guided coping strategies, or demonstrating behaviors that would represent risk to self or others) and physical (i.e., unstable vitals or any other medical concerns) and participant is not discharged until psychiatric and physical criteria are met. If they are assessed as ready for discharge, they are provided a list of potential adverse events to be aware of for the following 24 h, as well as the contact information for a 24 h phone number to contact study physician and the location of the closest emergency department. After discharge, two trained team members walk the participant across the street to a nearby hotel, and a trained team member conducts a follow up call approximately 1 h later to check in on how the participant is doing.

Each subsequent session (*Visits 3–10*, which do not involve drug administration) include conducting one imaginal exposure therapy session and processing of this experience. Homework is assigned, including listening to the tape of the imaginal exposure and reading the patient workbook. Skin conductance during all imaginal exposure therapy sessions is collected using the eSense application. Visit 3 includes both therapists and includes discussion of the MDMA experience, integration and planning for ongoing integration and self-care, and the third imaginal exposure and processing. In Visit 4, the in vivo exposure hierarchy is constructed, and the patient is instructed in *in vivo* exposure for homework at all subsequent sessions. Hot spots are introduced when clinically indicated, usually in Visit 5 or 6. All other sessions are staffed by the primary therapist except for the termination visit. Visit 10 includes recounting the entire memory, reviewing the in vivo hierarchy, discussing the experience for the patient including the role of the MDMA, success in reaching goals, future goals and continued work, termination, and leading a life characterized by approach rather than avoidance.

With regard to expected key aspects of treatment development, we have several questions we will focus on including participant's ability to engage in imaginal exposures while experiencing acute MDMA effects, what level and specific types of flexibility or adaptation with imaginal exposure and processing techniques may be indicated during MDMA session, optimal timing of two imaginal exposures during MDMA session, and strategies for approaching non-directive time during the MDMA session using an exposure based, “approach whatever arises” manner, and flexibility or content that may arise in subsequent PE sessions following an MDMA session.

Therapists. Therapists have PhDs in Clinical Psychology, are licensed psychologists, and are experts in delivering PE. All therapists for this study completed the MAPS [now Lykos] MDMA-AT program. The primary therapist meets with the participant for all therapy sessions. A secondary therapist meets with the participant and primary therapist for the first therapy session (i.e., the day before MDMA administration, “preparation”), the duration of the MDMA administration session (Visit 2, “dosing day”), and the therapy session the day after the MDMA administration (“integration”). It is optional for the secondary therapist to attend Visit 10 (termination) and any other sessions deemed clinically appropriate.

### 6.1 Assessment timepoints

During Visit 1, baseline assessments prior to exposure therapy are administered, including fear acquisition startle procedures, and psychological measures including the CAPS-5-R, the PTSD Checklist for DSM-5 (PCL-5), and the Patient Health Questionnaire 9-item version (PHQ-9). Post-treatment assessments, including all clinical measures, occur after Visit 10. Participants are contacted at one week, two weeks, and four weeks following treatment completion for follow-up symptom assessment, with primary outcome of CAPS-5-R at 4-week follow up.

### 6.2 Measures

Safety Screening. Study Visit 1 (V1) includes eligibility confirmation assessments and urine drug and pregnancy tests, electrocardiogram and 1 min rhythm strip, blood draw for screening and research labs, review of concomitant medications, physical exam, and vitals. Adverse affects will be assessed throughout study participation to assess overall safety. Clinical interviews. Primary outcome is change in The Clinician-Administered PTSD Scale for DSM-5 (CAPS-5-R) [29] total severity score from baseline to four-week follow-up time point. Psychophysiological Assessment. Participants complete the startle fear conditioning on Visit 1, extinction training session on Visit 3, followed by a startle fear extinction recall session on Visit 5, 2 days following the fear extinction session, and consistent with our previous methods [18]. In addition, a virtual reality startle session is conducted at pre- (Visit 1) and post-treatment (Visit 10) to assess physiological response to treatment. Startle Acquisition and fear extinction sessions are conducted at Visit 9 and a startle fear extinction recall session at Visit 10. While these analyses will be preliminary given the small sample size and lack of comparison arm, we will to investigate if MDMA + PE is associated with strong retention of extinction learning, and this preliminary data will inform methods for a subsequent placebo-controlled, sufficiently powered randomized trial. Self-Report Measures. The PCL-5 [30] and PHQ-9 [31] are secondary outcome measures to be assessed pre- and post-treatment. Other measures include Therapeutic alliance [32], mystical experiences questionnaire (MEQ^)^ [33]^,^ personality traits [34], positive and negative affect, and the use of nicotine, alcohol, and drugs at baseline and post-treatment to conduct exploratory analyses. Safety Measure. Lifetime and past 12 months version of the C-SSRS (Columbia Suicide Severity Rating Scale) [[25], [35]] are administered at the initial Screening visit. All subsequent administrations utilize the Since Last Visit version. Measures During Treatment. PCL-5 and PHQ-9 are administered at Visits 3, 5, 6, 8, & 10. The C-SSRS (since last visit) is administered at Visits 3 and 6. Mobile Skin Conductance Assessment. Skin conductance is assessed using the eSense skin conductance system (Mindfield Biosystems, Inc., Berlin, Germany) on an encrypted study iPad (iOS10 or later) continuously during all imaginal exposure sessions. Follow-up Measures. One week following V10, participants will have a phone-based follow-up call to administer the PCL-5, PHQ-9, and the CAPS-5, past week version. Two weeks following V10, participant completes PCL-5 and PHQ-9. Four weeks following V10, participant is contacted via phone call for a final assessment using the PCL-5, PHQ-9, CAPS-5 past one month version, C-SSRS and several self-report questionnaires.

Primary outcome: Primary outcome: The study primary outcome will be the CAPS-5-R PTSD dimensional severity score at 1 month follow up, with secondary outcomes of PCL-5 and PHQ-9. Statistical analysis will include one-way repeated measures analysis of variance (RM-ANOVA) and calculating Cohen's *d* effect size for changes. We expect significant decreases in CAPS-5-R, PCL-5, and PHQ-9 scores from baseline to 1 month post-treatment and a large effect size on reduction in PTSD symptom severity, as well as treatment acceptability and tolerability as assessed by participant completion of study methods and protocol.

## 7 Conclusions and next steps

MDMA in combination with therapy shows promise for PTSD treatment. This pilot study evaluates MDMA in combination with an evidence-based treatment, with significant translational support. Ultimately, this work will contribute to refinement of a manual of combining MDMA with exposure therapy, that subsequently can be disseminated and tested in a randomized, placebo-controlled trial.

## CRediT authorship contribution statement

**Jessica L. Maples-Keller:** Writing – review & editing, Writing – original draft, Supervision, Resources, Project administration, Methodology, Investigation, Conceptualization. **Boadie W. Dunlop:** Writing – review & editing, Supervision, Resources, Project administration, Methodology, Investigation, Funding acquisition, Conceptualization. **Barbara O. Rothbaum:** Writing – review & editing, Writing – original draft, Supervision, Resources, Project administration, Methodology, Investigation, Funding acquisition, Conceptualization.

## Relevant financial relationships

Dr. Maples-Keller has received consulting payments from COMPASS Pathways. Dr. Barbara Rothbaum has received Research/Grants from Wounded Warrior Project, NIH, Department of Defense, NSF, Bob Woodruff Foundation, MAPS; is a stockholder in Virtually Better; has been paid as a consultant by Otsuka, Psychwire, Senseye, MAPS, Jazz Pharmaceuticals, GoodCap, Transcend, Penumbra; serves on the Scientific Advisory Boards for Anxiety and Depression Association of America (ADAA), National Center for PTSD; serves on the Board of Directors for GratitudeAmerica; receives Royalties from Oxford University Press, APPI, Emory University. Emory Healthcare Veterans Program is supported by a grant from the Wounded Warrior Project. The terms of these arrangements have been reviewed and approved by Emory University in accordance with its conflict of interest policies. Dr. Dunlop has received research support from Boehringer Ingelheim, Compass Pathways, NIMH, and the Usona Institute, and has served as a consultant for Biohaven, Cerebral Therapeutics, Myriad Neuroscience, and Otsuka.

## Sources of funding

Funding Sources: The authors acknowledge, with gratitude, critical support from Wounded Warrior Project, who has supported this research and The Emory Healthcare Veterans Program (EHVP) and served as a partner in the Warrior Care Network-dedicated to filling gaps in mental health care for the invisible wounds of war in service members, Veterans and military families. Support was also received for the Multidisciplinary Association for Psychedelic Studies.

## Declaration of competing interest

The present research is partially funded by Multidisciplinary Association of Psychedelic Studies.
